# Bacterial Lipoprotein Posttranslational Modifications. New Insights and Opportunities for Antibiotic and Vaccine Development

**DOI:** 10.3389/fmicb.2021.788445

**Published:** 2021-12-07

**Authors:** Luke Smithers, Samir Olatunji, Martin Caffrey

**Affiliations:** ^1^School of Medicine, Trinity College Dublin, Dublin, Ireland; ^2^School of Biochemistry and Immunology, Trinity College Dublin, Dublin, Ireland

**Keywords:** antibiotic development, bacterial lipoproteins, lipoprotein processing enzymes, macromolecular X-ray crystallography, membrane proteins, posttranslational lipid modification, structure-based drug design, vaccine development

## Abstract

Lipoproteins are some of the most abundant proteins in bacteria. With a lipid anchor to the cell membrane, they function as enzymes, inhibitors, transporters, structural proteins, and as virulence factors. Lipoproteins activate the innate immune system and have biotechnological applications. The first lipoprotein was described by Braun and Rehn in 1969. Up until recently, however, work on lipoproteins has been sluggish, in part due to the challenges of handling proteins that are anchored to membranes by covalently linked lipids or are membrane integral. Activity in the area has quickened of late. In the past 5 years, high-resolution structures of the membrane enzymes of the canonical lipoprotein synthesis pathway have been determined, new lipoprotein types have been discovered and the enzymes responsible for their synthesis have been characterized biochemically. This has led to a flurry of activity aimed at developing novel antibiotics targeting these enzymes. In addition, surface exposed bacterial lipoproteins have been utilized as candidate vaccine antigens, and their potential to act as self-adjuvanting antigens is increasingly recognized. A summary of the latest developments in lipoproteins and their synthesis, as well as how this information is being exploited for therapeutic purposes is presented here.

## Introduction

The World Health Organisation has deemed antimicrobial resistance (AMR) one of the greatest threats to global health, food security, and economic development in the modern era (World Health Organisation, 2015). Infections by pathogenic organisms resistant to almost all known antibiotics are increasingly common (O’Neill, 2016; Zaman et al., 2017; Aslam et al., 2018; Jackson et al., 2018). Misuse of antibiotics in both the agriculture and healthcare sectors has hastened the development of microbial resistance. The appalling tardiness with which antimicrobials are being developed has exacerbated the situation, with no new antibiotics approved for use in over 50 years (Zaman et al., 2017; Aslam et al., 2018). This is attributed to the demanding safety requirements of regulatory bodies on novel therapeutics and the poor return on investment for pharmaceutical companies, with some abandoning antibiotic discovery programs altogether (Jackson et al., 2018). We now find ourselves approaching what has been referred to as a ‘post-antibiotic era,’ where once treatable infections, caused by something as simple as a scratch, may become a death sentence. Furthermore, without effective antibiotics, routine surgeries such as Cesarean sections, dental work, corrective joint procedures, and treatments that suppress the immune response, including chemotherapy, may prove too risky to perform (O’Neill, 2016).

Antimicrobial resistance poses a major threat, not only to health, but also to the economy. In 2016, an evaluation of the impact that AMR might have on the economy was carried out by United Kingdom economist, O’Neill (2016). The ensuing report indicated that by the year 2050, an estimated 10 million lives would be lost annually to AMR, at a cumulative cost of US$100 trillion. By comparison, the current coronavirus pandemic has resulted in less than 2.65 million deaths per year worldwide in the period December 2019 to November 2021 (Ritchie et al., 2020). Importantly, this is a rate that is in rapid decline thanks to the global administration of vaccines which is suppressing the severity of disease caused by SARS-CoV-2. Unfortunately, no such ‘quick fix’ is possible for AMR. Clearly, novel broad-spectrum antibiotics are urgently needed, and an emerging target for the development of new therapeutics is the bacterial lipoprotein posttranslational processing pathway.

Bacterial lipoproteins (BLPs) are a class of lipid-posttranslationally modified, membrane-anchored proteins that perform a variety of often essential functions (Kovacs-Simon et al., 2011). As the name implies, BLPs are exclusive to bacteria, with no equivalents in higher eukaryotes. BLPs are coded for by 1–5% of the bacterial genome (Babu et al., 2006). Some are transporters, importing various nutrients into the cell, while others, such as components of the acriflavin multidrug efflux pump, play a role in antibacterial resistance by exporting antibiotics out of the cell. BLPs can act as signaling molecules on the bacterial surface, they can aid in adhesion of bacteria to surfaces, and some are toxins (Babu et al., 2006). Braun’s lipoprotein (Lpp) is the most abundant protein in *E. coli* with up to a million copies per cell (Li et al., 2014). Once processed, mature Lpp is anchored to the inner leaflet of the outer membrane via its triacylated cysteine. The C-terminal region of the lipoprotein is covalently linked to the peptidoglycan layer in the periplasm, providing structural support to the cell wall and maintaining a relatively constant distance between the peptidoglycan layer and outer membrane.

Bacterial lipoproteins start out life as proteins anchored in the cytoplasmic membrane by a signal peptide. Subsequent lipidation and proteolysis generates the mature functional lipoprotein. The lipidation pattern at the N-terminus of BLPs, which act as potent agonists of Toll-like receptors (TLRs), identifies them as uniquely of bacterial origin and potentially harmful when detected by the innate immune system of higher organisms (Bessler et al., 1985; Takeuchi et al., 2001, 2002; Xia et al., 2021). Bacteria have thus evolved different lipidation profiles where TLR activation is altered in a way that presumably improves their chances of avoiding detection by the host’s immune system (Kurokawa et al., 2012; Nguyen and Götz, 2016; Nguyen et al., 2017; Armbruster et al., 2019, 2020). Owing to their many and varied essential functions, and the lack of homologous processing systems in humans, the BLP modification pathway is increasingly being recognized as a valuable target for the development of novel antibiotics.

In this review, we examine the enzymes involved in BLP synthesis and the potential use of lipoproteins and their processing enzymes as vaccines and drug targets. This work builds on previous reviews of BLPs and their processing enzymes to which the reader is referred (Buddelmeijer, 2015; Nguyen and Götz, 2016; Nguyen et al., 2020; Legood et al., 2021). We limit our analysis to BLPs that have an N-terminal cysteine or glycine lipid modification.

## Section 1: Enzymes of the BLP Processing Pathway

**FIGURE 1:**
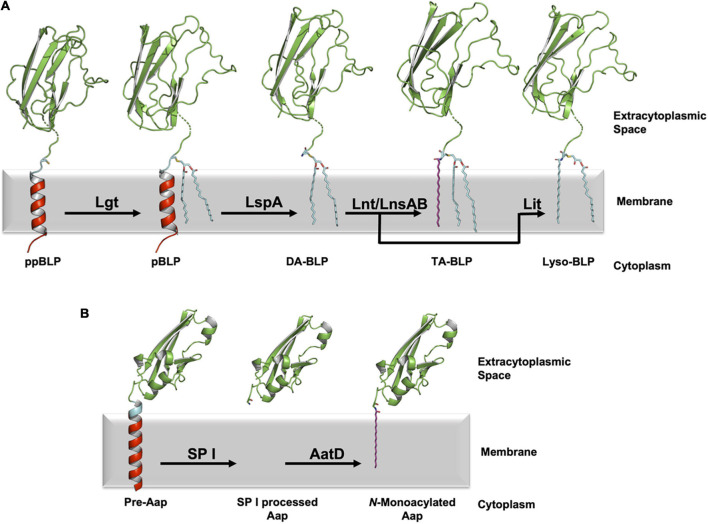
Processing of BLPs with N-terminal cysteine or glycine lipid modifications. **(A)** A newly synthesized ppBLP is shown on the left after being inserted into the cytoplasmic membrane. Its C-terminal U-domain from the Inhibitor of Cysteine Protease (ICP, from *Pseudomonas aeruginosa*, PDB code 2WGN) is shown as a green cartoon with the lipobox in cyan and the cysteine side chain as sticks. The remainder of the signal peptide is in red. The N-terminal portion of the BLP substrates and products in the figure were modeled in PyMol based on the sequence of ICP (UniProt code Q9I5G0), with the dashed breaks in the chain indicating where the modeling ends and the solved structure begins. For clarity, the acyl chains in the lipids and the lipidated BLPs are shown predominantly in the all-*trans* (fully extended/straight) conformation in this and subsequent figures. In reality, there is significant *cis*/*trans* isomerization along each chain such that they occupy approximately one half of the width of the lipid bilayer. The first reaction in the pathway is catalyzed by Lgt, which adds a diacylglyceryl moiety to the thiol group of the lipobox cysteine forming the pBLP product. pBLP acts as substrate for LspA, which cleaves the signal peptide to the N-terminus of the lipobox cysteine forming a diacylated BLP (DA-BLP) product. Lnt catalyzes the addition of a third acyl chain to the N-terminal amine of the DA-BLP forming a triacylated BLP (TA-BLP). In low GC Gram-positive Firmicutes this third step is catalyzed by the LnsAB complex. In low-GC Gram-positive Firmicutes, Lit catalyzes the intramolecular transfer of an acyl chain at the *sn*-2 position of the diacylglyceryl moiety in the DA-BLP to its N-terminal amine forming a lyso-BLP. **(B)** A different class of lipoproteins that are acylated at an N-terminal glycine residue has recently been identified in pathogenic strains of *E. coli*. The bacterial surface protein, dispersin (Aap) is one such lipoprotein. Immature dispersin (Pre-Aap, AlphaFold2 model) is shown inserted in the cytoplasmic membrane with its signal peptide colored red, its signal peptidase I cleavage motif (AXA) colored blue and its U-domain green. The signal peptide is cleaved by signal peptidase I (SP I) after which AatD acylates the soluble U-domain at the α-amino group of its N-terminal glycine to anchor the protein in the membrane.

In the canonical pathway, posttranslational modifications of BLPs are carried out by two or three integral membrane enzymes in Gram-positive and Gram-negative bacteria. The nascent BLP is translated in the cytoplasm as a preprolipoprotein (ppBLP). The ppBLP consists of an N-terminal signal peptide that is on average 16–26 residues long (Babu et al., 2006) and a C-terminal functional or ultra-domain (U-domain). The signal peptide has three recognizable parts: an N-terminal positively charged sequence, a hydrophobic helix (h-region), and a C-terminal lipobox. The latter consists of a highly conserved sequence of form: [LV]^–3^ [ASTVI]^–2^ [GAS]^–1^ [C]^+1^, where the lipobox cysteine is invariant and is the site of lipidation (Babu et al., 2006). Separating the U-domain and the lipobox cysteine is a tether, or linkage region of varying length that is typically disordered (Roszak et al., 2012; Wiktor et al., 2017; El Rayes et al., 2021). The signal peptide is inserted into the cytoplasmic membrane by means of the Secretory (Sec) (unfolded functional domains) or the Twin-Arginine Translocation (TAT) (folded functional domains) pathways, with the lipobox positioned proximal to the extracytoplasmic membrane surface (Hayashi and Wu, 1985; Watanabe et al., 1988; Kosic et al., 1993; De Buck et al., 2004; Fröderberg et al., 2004; Widdick et al., 2006; Giménez et al., 2007; Shruthi et al., 2010; Thompson et al., 2010). It is here that the sequential posttranslational modifications catalyzed by lipoprotein diacylglyceryl transferase (Lgt), lipoprotein signal peptidase (LspA) and lipoprotein *N*-acyl transferase (Lnt) take place (Figure 1).

In Gram-negative bacteria, fully processed BLPs can traffic to the outer membrane via the localization of lipoprotein (Lol) pathway. This pathway has been reviewed extensively elsewhere (Tokuda and Matsuyama, 2004; Okuda and Tokuda, 2011; Tokuda et al., 2014; Zückert, 2014; Narita and Tokuda, 2017). Briefly, it consists of an ATP-binding cassette transporter (LolCDE complex) that resides in the inner membrane, a periplasmic chaperone or carrier protein (LolA) and an outer membrane receptor (LolB), which itself is a BLP. The identity of residues that follow the fully lipidated N-terminal cysteine in the mature BLP has been shown to play a role in determining whether it is trafficked by the Lol pathway to the outer membrane or is retained in the inner membrane (Yamaguchi et al., 1988; Gennity and Inouye, 1991; Terada et al., 2001; Narita and Tokuda, 2006; Lewenza et al., 2008). Routing decisions may also be made based on the character, such as the disorder and length of the tether region of the mature BLP (El Rayes et al., 2021). Complete processing of BLPs in Gram-negative species is seemingly required for their correct localization. This has been proposed as the reason for the essentiality of the enzymes that carry out the posttranslational modifications in these bacteria (Malinverni et al., 2006; Wu et al., 2006; Misra et al., 2015).

In Gram-positives, which lack an outer membrane, all BLPs localize to the outer leaflet of the cytoplasmic membrane and thus, do not require trafficking (Nguyen and Götz, 2016). As a result, posttranslational modification is not always necessary (Petit et al., 2001; Sander et al., 2004; Wardenburg et al., 2006; Buddelmeijer, 2015; Nguyen and Götz, 2016). In Gram-positive bacteria, the immature ppBLP, anchored in the membrane by way of its N-terminal signal peptide, is presumably functional. However, many Gram-positive pathogenic organisms, including *Mycobacterium tuberculosis, Staphylococcus aureus*, and *Streptococcal* species, show attenuated virulence when posttranslational modifications, catalyzed by Lgt and LspA, are compromised (Petit et al., 2001; Sander et al., 2004; Stoll et al., 2005; Khandavilli et al., 2008; Bray et al., 2009; Chimalapati et al., 2012).

The first three enzymes involved in the posttranslational modification of lipoproteins considered in this review are members of the canonical BLP posttranslational pathway. The last three are enzymes only recently identified.

### Lipoprotein Diacylglyceryl Transferase

**FIGURE 2:**
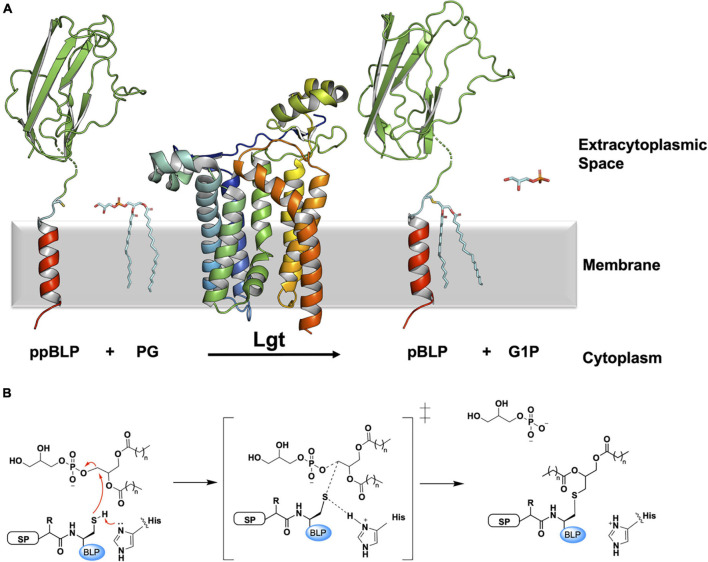
Reaction scheme for the BLP posttranslational modification carried out by Lgt. **(A)** The reaction catalyzed by Lgt is illustrated using the structure of Lgt (PDB code 5AZB). The substrates and products are colored as in Figure 1. Lgt (shown in cartoon representation colored blue to red from N- to C-terminus) catalyzes the transfer of diacylglyceryl preferentially from phosphatidylglycerol (PG) to the thiol group of the lipobox cysteine. The products of the reaction include pBLP and glycerol-1-phosphate (G1P). **(B)** A putative mechanism for the reaction catalyzed by Lgt, adapted from Singh et al. (2019). Left: Michaelis complex displaying parts of the ppBLP substrate adjacent to the catalytic histidine and the PG substrate. Red curved arrows indicate electron flow. Electron lone pairs are shown as double dots. SP, signal peptide; BLP, tether and U-domain of the BLP; His, catalytic histidine. Middle: reaction intermediate. Right: pBLP and G1P products alongside the protonated form of the catalytic histidine of Lgt.

Lgt catalyzes the first lipid-posttranslational modification in the pathway, converting the ppBLP to a prolipoprotein (pBLP) (Sankaran and Wu, 1994). As noted, Lgt is essential in Gram-negative bacteria and is often required for virulence in Gram-positive species (Petit et al., 2001; Stoll et al., 2005; Malinverni et al., 2006; Wu et al., 2006; Bray et al., 2009; Chimalapati et al., 2012; Misra et al., 2015). Lgt recognizes and binds the signal peptide of an incoming ppBLP substrate and catalyzes the formation of a thioether link between the thiol group on the invariant lipobox cysteine, and a diacylglyceryl moiety from a lipid substrate, primarily phosphatidylglycerol (PG). The pBLP product of Lgt is doubly anchored in the cytoplasmic membrane by its signal peptide and the two fatty acyl chains in the diacylglyceryl moiety (Figure 2A).

Two X-ray crystal structures of Lgt from *Escherichia coli* (291 residues) have been solved (Mao et al., 2016). These have proven to be extremely useful in rationalizing earlier biochemical and biophysical work carried out on this integral membrane enzyme (Sankaran and Wu, 1994; Sankaran et al., 1997; Pailler et al., 2012). One of the structures, at a resolution of 1.6 Å, has a fatty acid, palmitic acid, and a detergent (*n*-octyl-β-D-glucoside), along with two PG molecules bound to the enzyme. The fatty acid and detergent molecules are referred to by the authors as inhibitors of the enzyme, while the PG molecules are at sites suggesting they contribute only to crystal packing. When considering this structure, the Reader should be mindful of the unexplained positive difference density around the putative catalytic histidine residue (His103). The second structure, at 1.9 Å resolution, was free of structured adventitious inhibitors and has a PG molecule in the proposed active site. Electron density attributed to a diacylglyceryl moiety was also found in the core of the enzyme. It was interpreted as having derived from PG and was used to demark a second lipid substrate binding pocket in Lgt.

**FIGURE 3:**
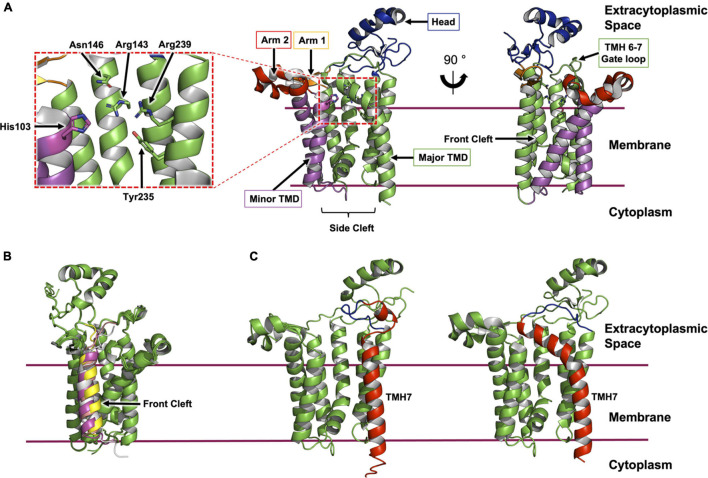
Structural features of Lgt from *E. coli.* **(A)** The structure of Lgt shown was created using PDB record 5AZB (Mao et al., 2016). The transmembrane domain (major domain, green; minor domain, magenta) is depicted bounded by two horizontal lines which approximate the limits of the cytoplasmic membrane. The location of the expansive side cleft between the two TMH domains is indicated below the structure. The two periplasmic arms are shown in orange and red with the periplasmic head domain in blue. Two different perspectives are shown. The first (middle panel) is a ‘side’ view from the membrane plane. The second (right panel) is a ‘front’ view with the front cleft indicated by an arrow. A close-up of the active site is shown (left panel) as indicated by the dashed red box. **(B)** ColabFold model of Lgt from *E. coli* (green) in complex with four ppBLP substrates (Lpp, magenta; Pal, yellow; LolB, gray; BamD, pink). For clarity, only the signal peptide, the lipobox and the next three amino acids of the ppBLP substrates are shown; the tether and U-domain are hidden. The front cleft, where all ppBLPs are predicted to bind is indicated. **(C)** A comparison of TMH7 (red) and the periplasmic gate loop (blue) in the AlphaFold2 model (left) and the X-ray crystal structure (right) of Lgt. It may be that the two structures represent, respectively, the enzyme with the periplasmic gate open and closed.

The enzyme consists of a bundle of seven transmembrane helices (TMH, Figures 2A, 3A). The cytoplasmic side of the bundle is cationic, consistent with the ‘inside-positive’ rule, and is where the C-terminus resides. The periplasmic side is described as consisting of a head domain, at the base of which are two amphiphilic extensions, one α-helical, the other composed of two antiparallel β-strands. These extend from the body of the protein like two out-stretched arms roughly parallel to the membrane interface. The TMH body has a minor TM domain consisting of TMH2 and TMH3, and a major domain consisting of TMH1 and TMH4-7. The major domain, with its five TMHs arranged like fingers on a hand, forms a curved surface into which the minor domain sits. Three functional features of the enzyme are proposed to situate along the interface between these two domains. At the core resides a central cavity decorated by conserved amino acids that are proposed to accommodate the active site. On either side of this cavity are clefts, or fenestrations, known as the front and side cleft that provide access and egress for substrates and products as they exchange between the active site, the rest of the membrane, and the periplasm (Figure 3A).

The side cleft between the major and minor TMH domains is the site of the highly conserved His^103^-Gly-Gly-Leu^106^ motif in Lgt from *E. coli*. Mutating His103 to asparagine or glutamine was previously shown to inactivate the enzyme (Sankaran et al., 1997). This led to the hypothesis that His103 is involved in catalysis (Figure 2B). Specifically, it is proposed to abstract a proton from the thiol of the lipobox cysteine on the ppBLP substrate generating a reactive thiolate. This nucleophile attacks the lipid substrate at the ester bond between the phosphate and diacylglyceryl moiety generating pBLP and glycerol-1-phosphate as products. Of note is the recent observation that Lgt from *E. coli* can use PG as a substrate in which the terminal glycerol is racemic at C2 (Diao et al., 2021). The enzyme can therefore produce both glycerol-1-phosphate and glycerol-3-phosphate as products. This feature has been exploited to provide a useful luciferase coupled assay for Lgt.

Based on the position of bound lipids and conserved residues in the structures of the enzyme, two mechanisms were originally advanced for how Lgt carried out its diacylglyceryl transferase reaction (Mao et al., 2016). The first has the enzyme preloaded with two PG molecules. The ppBLP substrate docks onto the side cleft near the catalytic His103, it reacts with one of the PG molecules and the product pBLP undocks into the membrane. The second PG molecule is proposed to ratchet into the space created by the departing pBLP while another PG replaces it from the membrane reservoir. The water-soluble glycerol-1-phosphate product diffuses into the periplasm. In the second model, a single PG molecule is bound to the enzyme away from the catalytic center, which is proposed to be blocked by way of a gate formed by a small loop between the periplasmic ends of TMH6 and TMH7 (Figure 3A). Interaction with the ppBLP substrate opens the gate, enabling the lipid and the BLP substrate to come together at the active site for reaction. The pBLP departs by the same route the ppBLP substrate entered while the glycerol-1-phosphate product diffuses into the periplasm.

Molecular dynamics simulations using the X-ray crystal structures of Lgt suggested an alternative reaction progression (Singh et al., 2019). Based on the hypothesized reaction mechanisms discussed above, the authors attempted to dock a short signal peptide and lipobox sequence at the side cleft of Lgt with a PG molecule in the active site. Over the course of a simulation, the ppBLP mimetic moved, on average, 12 Å away from the PG substrate, and the lipobox cysteine did not interact with the proposed catalytic His103 on Lgt. However, when the ppBLP mimetic was docked at the front cleft of the enzyme, it moved into the active site over the course of a 150 ns simulation. The entry of the ppBLP into the active site was accomodated by a series of conformational changes that included an opening of the gate formed by the periplasmic loop between TMH6 and TMH7 discussed above, which, in this model, is thought to enable the pBLP product to egress through the side cleft. At the end of the simulations, the average distance between the thiol group of the lipobox cysteine and the ester bond between the phosphate and diacylglyceryl moiety of the docked PG molecule was 3.5 ± 0.3 Å. The distance between the catalytic His103 and the thiol group of the lipobox cysteine of the ppBLP mimetic was 4.0 ± 0.5 Å. The authors surmised, based on these observations, that the reaction proceeds as follows. A PG substrate binds within the active site. The ppBLP substrate binds at the front cleft, away from the catalytic histidine. It then moves into the active site, close to the catalytic histidine and the PG substrate. This is accompanied by a series of conformation changes in the enzyme that includes opening the periplasmic gate. The reaction occurs as outlined in Figure 2B and the pBLP product departs through the side cleft, accommodated by the aforementioned gate opening. The glycerol-1-phosphate product diffuses into the periplasm.

Separately, we have modeled complexes of Lgt from *E. coli* with four different native *E. coli* lipoprotein substrates (LolB, Uniprot ID P61320; BamD, Uniprot ID P0AC02; Pal, Uniprot P0A912; Lpp, Uniprot ID P69776) using the recently released ColabFold protein structure prediction platform (Figure 3B). This freely available Google Colab notebook combines AlphaFold2 and RoseTTAfold with a fast multiple sequence alignment algorithm (Mirdita et al., 2021). In all cases, the software modeled the signal peptide at the front cleft, as was observed in the molecular dynamics simulations just described. It is interesting to note the form of TMH7 and the location of the periplasmic loop gate in the ColabFold and AlphaFold2 predicted models. In both, TMH7 is straight with the loop positioned well above the membrane surface. By contrast, in the crystal structures, TMH7 bends sharply with its N terminal end and the loop gate oriented toward the active site (Figure 3C). These disparate conformations may be interpreted as supporting the proposed role of the periplasmic loop between TMH6 and TMH7 as a gate. Thus, the experimental and predicted models correspond to the closed and open conformations, respectively. Modeling Lgt in complex with all members of the *E. coli* lipoproteome individually, of which there are between 80 and 120 depending on the strain, may help to explain how so many distinctly different substrates are able to bind, and to be processed by a single enzyme. Validation of such models with experimental methods such as X-ray and electron crystallography, single particle cryo-electron microscopy and nuclear magnetic resonance, will be required eventually. In so doing, the expectation is that the reaction mechanism and substrate selectivity of Lgt will be deciphered in atomic detail.

Several other residues have been identified as crucial for efficient diacylglyceryl transferase catalysis by Lgt in *E. coli*. These include Arg143, Asn146, Tyr235, and Arg239 (Figure 3A). These residues decorate the internal wall of the active site on the major TM domain. Mutations at these sites resulted in either a complete loss of activity (Asn146Ala, Tyr235Phe, Tyr235Thr) or a significant reduction in activity (Arg143Ala, Tyr235Ser, Arg239Ala) (Sankaran et al., 1997; Pailler et al., 2012). *In silico* docking of a PG substrate molecule into the proposed active site provided further insight into the function of these important residues (Singh et al., 2019). The docked lipid was so positioned to replace the diacylglyceryl observed in the 1.9 Å crystal structure of Lgt (PDB ID 5AZC). Here, a series of complex hydrogen bond networks (involving residues Arg143, Asn146, Ser198, Glu202, Tyr235, Arg239, Arg246, and four coordinated waters) accommodate both the phosphate moiety and glycerol head group of the PG substrate in an otherwise apolar recess. The previously established essentiality of these residues is therefore presumably due to their role in coordinating the polar head group of PG within the hydrophobic active site over the course of the reaction.

The pBLP produced as a result of the diacylglyceryl transfer reaction catalyzed by Lgt serves as a substrate for the second enzyme in the canonical pathway, lipoprotein signal peptidase, which is described next.

### Lipoprotein Signal Peptidase

**FIGURE 4:**
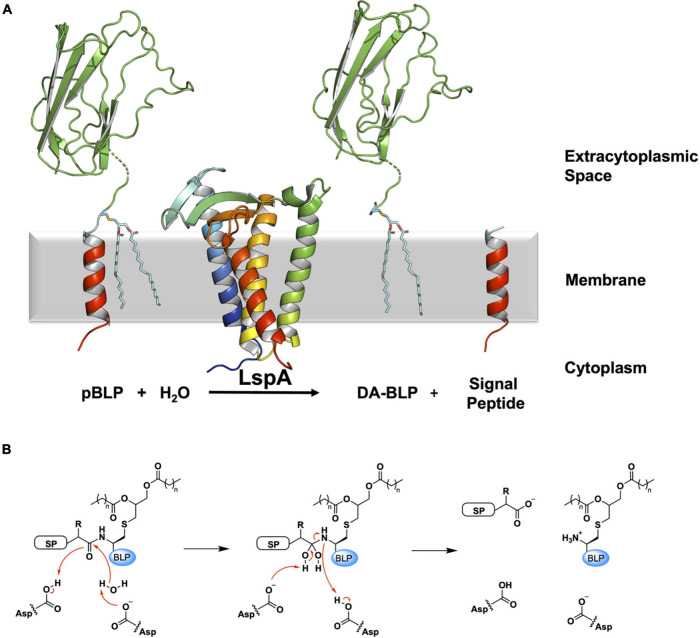
Reaction scheme for the BLP posttranslational modification carried out by LspA. **(A)** The reaction catalyzed by LspA is illustrated using structures of LspA from *S. aureus* (PDB code 6RYO) and of the U-domain of ICP (PDB code 2WGN), a BLP from *P. aeruginosa*. LspA recognizes the lipidated BLP, pBLP, and cleaves off the signal peptide to the N-terminus of the lipobox cysteine with the aid of a catalytic water molecule. This generates the DA-BLP, and a free signal peptide as the second product of the reaction. Coloring and details for LspA and the BLP substrate and products are as in Figure 1. The natural antibiotic, globomycin, which occupies the enzyme active site in the crystal structure of LspA, is not displayed for clarity. **(B)** A putative mechanism for the reaction catalyzed by LspA, adapted from Vogeley et al. (2016). Left: Michaelis complex displaying parts of the pBLP substrate around the scissile bond. SP, signal peptide; BLP, BLP tether and U-domain; Asp, catalytic aspartates. Middle: Tetrahedral intermediate. Right: DA-BLP product and regenerated catalytic aspartates. Red curved arrows indicate electron flow.

LspA carries out the second step in the BLP processing pathway. It cleaves the signal peptide to the N-terminal side of the lipidated cysteine. In so doing, LspA generates a free signal peptide, and a diacylated-BLP (DA-BLP) that is now anchored in the membrane by the two fatty acyl chains of its diacylglyceryl moiety (Figure 4).

**FIGURE 5:**
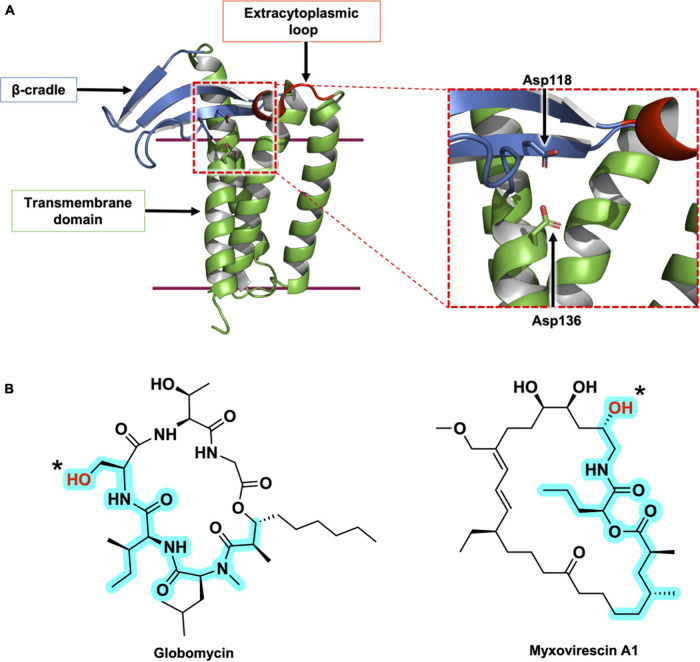
Structural features of LspA and its natural inhibitors, globomycin and myxovirescin. **(A)** The structure of LspA from *S. aureus* (PDB 6RYO) is shown in cartoon representation. The three main structural domains are colored with the β-cradle in blue, the extracytoplasmic loop in red and the transmembrane domain in green. The active site is indicated with a dashed red box. A magnified view of the active site is presented on the right. The two catalytic aspartate residues are shown in stick representation. The globomycin inhibitor which occupies the active site in the crystal structure has been removed for clarity. **(B)** Structures of the natural antibiotics, globomycin and myxovirescin. The blocking hydroxyl in both inhibitors is identified with an asterisk. The 19-atom backbone motif that interacts with LspA and that is common to the two antibiotics is highlighted in cyan.

The *lspA* gene was identified originally by Inukai et al. (1978), in their study of globomycin, a 19-membered cyclic depsipeptide antibiotic produced by *Streptomyces* species (Figure 5A). Globomycin inhibits the growth of certain Gram-negative bacteria such as *E. coli* and *K. pneumoniae*. Interestingly, the depsipeptide is inactive against *Pseudomonas* and Gram-positive bacteria (Inukai et al., 1978). While speculative, reasons for this observation include an outer membrane with reduced permeability to globomycin in the case of *Pseudomonas sp.*, and a lack of Lpp toxicity due to mislocalization in the case of Gram-positive organisms (discussed below). Further investigation is required to fully explain these findings.

Another natural product inhibitor of LspA is the 28-membered macrolactam lactone, myxovirescin, produced by *Myxococcus xanthus* (Rosenberg et al., 1973; Rosenberg and Dworkin, 1996; Xiao et al., 2012). The globomycin and myxovirescin producing bacteria harbor multiple copies of the *lspA* gene. *Streptomyces hagronensis* has two copies of *lspA*, while *Myxococcus xanthus* has four *lspA* paralogs (*lspA1 – 4*). *lspA1* and *lspA2* are located in tandem within the genome, in the same operon as *lgt*, and are proposed to perform housekeeping peptidase functions in processing the lipoproteins of *M. xanthus* (Xiao and Wall, 2014). In contrast, *lspA3* and *lspA4* belong to the myxovirescin biosynthetic gene cluster. In addition to the peptidase activities of these two orthologs, they are thought to play a role in regulating the levels of myxovirescin produced by *M. xanthus* thereby protecting the bacteria against its own toxic natural product (Xiao and Wall, 2014).

Interestingly, none of the four LspAs from *M. xanthus* could rescue cell viability of an *E. coli lspA* depletion mutant in the presence of the highly abundant Braun’s lipoprotein, Lpp (Xiao and Wall, 2014). It is hypothesized that these four LspAs are less efficient than their *E. coli* counterpart in processing BLPs, resulting in the toxic accumulation of the ppBLP and pBLP forms of Lpp in the inner membrane. Indeed, when *lpp* is deleted, the four LspAs from *M. xanthus* can complement the *E. coli lspA* depletion mutant, rendering the cells viable. This confirms they all have peptidase activities but are less efficient than their *E. coli* homolog. The lower peptidase activity of *M. xanthus* LspAs in *E. coli* can potentially be explained by a reduced translation efficiency of the enzyme, resulting in a lesser amount of expressed LspAs, different substrate specificities, and/or by lower intrinsic enzyme activities (Xiao and Wall, 2014).

The X-ray crystal structures of LspA from *P. aeruginosa* (169 amino acids) and *S. aureus* (163 amino acids) in complex with globomycin have been solved (Vogeley et al., 2016; Olatunji et al., 2020). The structures reveal a membrane domain consisting of four TMHs and an extracytoplasmic domain composed of two motifs, a β-cradle and a loop with a single-turn helix. The N- and C-termini are in the cytoplasm, and consistent with the positive inside rule, the cytoplasmic end of the TMH bundle is predominantly cationic (Figure 5A). Both the β-cradle and extracytoplasmic loop are proposed to interact with residues in the tether and possibly the U-domain of the pBLP substrate (Vogeley et al., 2016).

A comparison of LspA sequences from different bacterial species identifies 14 highly conserved residues, including two strictly conserved aspartates. They cluster to the extracytoplasmic end of the TMH bundle where it contacts the β-cradle and the one-turn helical loop. Studies assessing the activity of LspA upon mutation of these two aspartates support their essential and catalytic roles. The enzyme is proposed to function as an aspartyl protease (Tjalsma et al., 1999; Vogeley et al., 2016; Olatunji et al., 2020). A putative mechanism for how LspA catalyzes the peptide cleavage reaction is included in Figure 4B. Endopeptidolysis begins with the abstraction of a proton from water by one of the aspartyl residues in the catalytic dyad. The hydroxide nucleophile so generated attacks the carbonyl carbon in the scissile peptide bond. The nucleophilic oxygen at that carbon abstracts a proton from the second catalytic aspartyl residue and, in so doing, forms a tetrahedral intermediate. A proton is transferred from the first aspartyl residue to the amide nitrogen at the core of the tetrahedral intermediate. The scissile bond is then cleaved when a proton returns to the second aspartyl to release the free signal peptide and the DA-BLP.

The crystal structures of LspA from *S. aureus* complexed with globomycin and myxovirescin reveal a similar binding pose for the two antibiotics (Olatunji et al., 2020). Both compounds have, what has been referred to as, a blocking hydroxyl group which sits between and within 3 Å of the two catalytic aspartates in the complex structures. So situated, the antibiotics block the active site and inhibit the enzyme. Strikingly, globomycin and myxovirescin share a common 19-atom backbone motif, at one end of which is the blocking hydroxyl (Figure 5B). The polar backbone or spine extends away from the catalytic center toward the cytoplasmic side of the enzyme in a way that mimics the lipobox residues in the proposed LspA-pBLP Michaelis complex. The remainder of the antibiotics’ more hydrophobic cyclic structure occupy opposite sides of the substrate binding pocket; the depsipeptide ring of globomycin sits oriented toward the right side of the pocket (looking into the binding pocket from the membrane plane with the cytoplasmic end down) while the myxovirescin macrocyclic lactone locates to its left side. In so doing, these amphiphilic molecules effectively dock into and block a polar binding pocket of the enzyme that is situated within the lipid membrane or proximal to the membrane interface where peptidolysis takes place. Structure-based design of drugs targeting active sites that are accessed from the membrane has emerged recently as an area of particular interest for medicinal chemists (Payandeh and Volgraf, 2021).

In some cases, the DA-BLP product produced by LspA is the final, fully mature form of the lipoprotein. In others, the DA-BLP serves as substrate for the third and final posttranslational modifying enzyme in the canonical pathway, apolipoprotein *N*-acyl transferase, which is introduced next.

### Lipoprotein *N*-acyl Transferase

**FIGURE 6:**
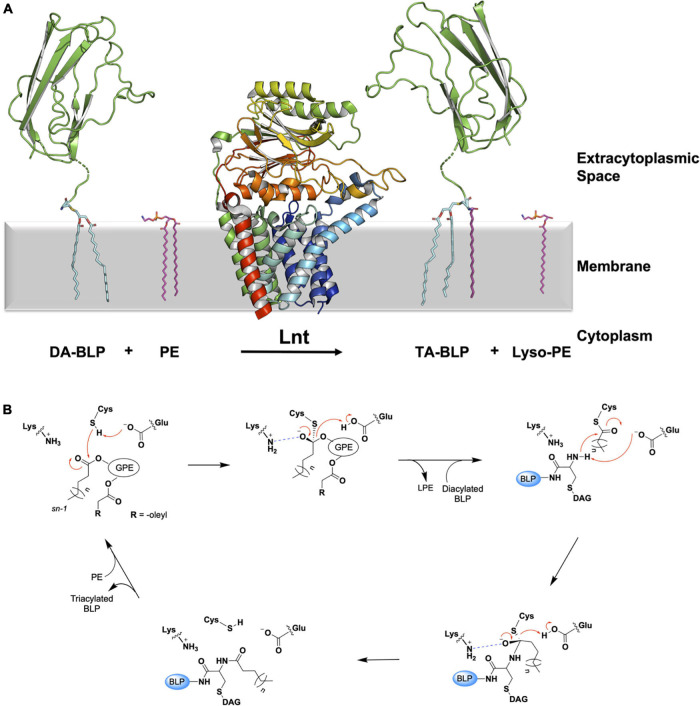
Reaction scheme for the BLP posttranslational modification carried out by Lnt. **(A)** The reaction catalyzed by Lnt is illustrated using structures of Lnt from *E. coli* (PDB code 5N6H) and of the U-domain of ICP (PDB code 2WGN), a BLP from *P. aeruginosa*. Lnt first binds phosphatidylethanolamine (PE, purple sticks) and uses the acyl chain at the *sn-*1 position of the lipid substrate to acylate itself. The lyso-PE product leaves the active site to be replaced by the DA-BLP substrate. Lnt then transfers its acyl chain to the DA-BLP substrate, forming the TA-BLP product. **(B)** A putative mechanism for the reaction catalyzed by Lnt adapted from Wiktor et al. (2017). Top left: First Michaelis complex with the PE substrate. Top middle: First tetrahedral intermediate. Tetrahedral carbon shown in stereochemical representation. The oxyanion bears a negative charge. Top right: Second Michaelis complex between the acylated Lnt and a DA-BLP substrate. Bottom right: Second tetrahedral intermediate. Bottom left: Lnt-product complex. Electron lone pairs are shown as double dots. Red curved arrows indicate electron flow. Dashed blue lines denote oxyanion stabilization. GPE, glyceryl-phosphoethanolamine; BLP, tether and U-domain of the lipoprotein; DAG, diacylglyceryl; LPE, lyso-PE.

Lnt catalyzes the transfer of an acyl chain, preferentially from phosphatidylethanolamine (PE) to the free ammonium group on the lipidated N-terminal cysteine of DA-BLP formed by LspA. The product of the Lnt reaction is now a triacylated-BLP (TA-BLP). The Lnt catalyzed reaction is proposed to follow a ping-pong mechanism. The enzyme first binds PE. It then cleaves the acyl chain at the *sn*-1 position on glyceryl in the lipid and acylates itself via a thioester linkage to the catalytic cysteine (Cys387 in *E. coli*). The lyso-PE product exits the active site before the DA-BLP substrate enters, whereupon the acyl chain on the enzyme is transferred to the N-terminal ammonium group of the BLP substrate (Figure 6).

**FIGURE 7:**
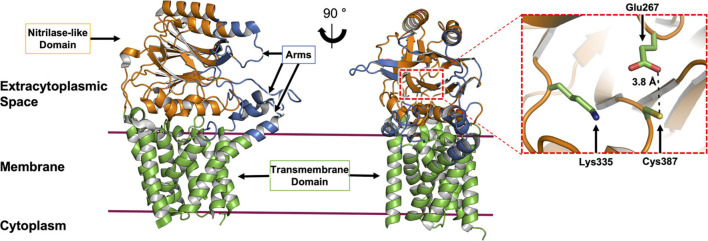
Structural features of Lnt. The structure of Lnt from *E. coli* (PDP code 5N6H) is shown. Side **(left)** and front views **(right)** of the enzyme. The nitrilase-like domain (orange), transmembrane domain (green) and arms (blue) are indicated. The catalytic site (dashed red box in front view) is shown magnified (right). The catalytic triad that includes Glu267, Lys335 and Cys387, in the nitrilase-like domain is shown colored by element, with carbon atoms in green, nitrogen in blue, oxygen in red and sulfur in yellow. The distance between Glu267 and Cys387 is shown. Glu267 is proposed to initiate the reaction by abstracting a proton from the thiol at Cys387.

To date, nine X-ray crystal structures of Lnt are available (Lu et al., 2017; Noland et al., 2017; Wiktor et al., 2017; Wiseman and Högbom, 2020; van Dalsen et al., 2021). These derive from wild type *E. coli* (Lnt*Eco*, 512 residues) and *P. aeruginosa* (Lnt*Pae*, 511 residues), and include structures of inactive mutant forms of the *E. coli* enzyme (Cys387Ala and Cys387Ser). The protein has two recognizable parts, a transmembrane domain, consisting of eight TMHs and a periplasmic nitrilase-like domain. The latter includes the αββα sandwich motif of the nitrilase superfamily. The active site, where the catalytic triad (Cys, Glu, Lys) is located, is housed in the nitrilase-like domain, approximately 13 Å above the extracytoplasmic surface of the inner membrane. A series of loops, referred to as arms, extend from the nitrilase-like domain, as well as from extensions to the transmembrane helices. The arms run roughly parallel to the membrane surface and create a funnel at the narrow end of which sits the catalytic triad. These loops or arms are speculated to guide the lipid and BLP substrates and products into and out of the active site and may play a gating role (Figure 7). Molecular dynamics simulations support the hypothesis that the funnel is the lateral point of entry and egress for both substrates and products (Wiktor et al., 2017).

The available structures are of the substrate-free form of the enzyme. Some have monoolein molecules in the funnel and active site. Monoolein is the lipid used to grow crystals for structure determination by the *in meso* or lipid cubic phase method (Caffrey, 2015). The lipid-bound structures have been likened to the Lnt-PE Michaelis complex proposed to form as part of the ping-pong reaction mechanism. While this is a reasonable interpretation, there are significant structural and chemical differences between monoolein and PE molecules that warrant the pursuit of a true PE-bound structure. Relatedly, the structure of Lnt*Eco* acylated at its catalytic cysteine has been reported (PDB ID: 6NWR, Wiseman and Högbom, 2020). Being able to capture an acyl-Lnt intermediate structure comes as a surprise given the typically labile nature of the thioester linkage between a cysteine and a fatty acyl chain. While this reported structure should provide useful information regarding enzyme-intermediate interactions within the active site, other important regions of the protein, one of the funnel arms in particular, engage in crystal contacts. These can induce conformations whose physiological relevance deserves careful consideration.

The Lnt transacylation reaction is proposed to take place at the catalytic triad comprising Glu267, Cys387, and Lys335 (residue numbering in *E. coli*) as outlined in Figure 6B. Upon binding an appropriate phospholipid substrate in the active site, preferentially PE, Glu267 abstracts a proton from the sulfhydryl group on the side chain of Cys387 to generate a nucleophilic thiolate. The thiolate is then free to attack the ester linkage between the glyceryl and acyl chain at the *sn*-1 position in the PE head group. This leads to the formation of the first tetrahedral intermediate. Lys335 acts to stabilize the negatively charged oxyanion on the intermediate by creating an oxyanion hole with the aid of backbone amides from Ile390, Ile391, and Leu392. The oxyanion attacks the tetrahedral carbon, which in turn abstracts a proton from Glu267, forming the acyl-Lnt intermediate and lyso-PE. The lyso-lipid product leaves the active site, making way for the DA-BLP substrate to enter for the second half of the reaction to begin. The α-amino group at the N-terminus of the newly arrived lipoprotein attacks the carbonyl carbon of the acylated cysteine in the acyl-Lnt intermediate, forming the second tetrahedral intermediate. The resulting oxyanion is stabilized once again by the oxyanion hole formed by Lys335, Ile390, Ile391, and Leu392. Collapse of the second intermediate generates the TA-BLP product which leaves the active site to regenerate Lnt for another round of catalysis.

While Lgt and LspA are highly conserved across a wide range of prokaryotes, Lnt is considerably less so. Lnt is absent in low-GC Gram-positive bacteria and is not essential in high-GC Gram-positive species or *Mycobacteria*. Furthermore, Lnt is not always essential in Gram-negative organisms (Vidal-Ingigliardi et al., 2007; Tschumi et al., 2009; LoVullo et al., 2015). A major outcome of triacylation by Lnt appears to be recognition by the Lol trafficking system for transfer of the TA-BLP across the periplasm to the outer membrane. As noted, all Gram-positive BLPs localize to the outer leaflet of the cytoplasmic membrane and therefore do not require trafficking. This perhaps explains why Lnt is dispensable, or indeed absent in monoderms.

With regards to Gram-negative diderms, it has been shown that *lnt* deletions in most species, including *E. coli*, results in toxic accumulation of BLPs in the inner membrane that would otherwise be trafficked to the outer membrane (Gupta et al., 1993; Robichon et al., 2005). This ultimately results in a loss of cell viability. Cells can be rescued by overexpression of LolCDE, suggesting that the Lol trafficking system can indeed transport DA-BLPs but with reduced efficiency compared to TA-BLPs (Narita and Tokuda, 2011). Conversely, *lnt* deletions are not lethal in certain Gram-negative diderms, such as *Neisserial* sp., *Helicobater pylori*, and *Acinetobacter* sp. (LoVullo et al., 2015). This has been proposed to originate from a slight alteration to the Lol trafficking system in these species. Here, an alternative ABC-transporter transmembrane component, LolF, which forms a homodimeric transmembrane domain, as opposed to the more common heterodimeric LolCE complex is present. It has been proposed that the LolF homodimeric TMD can recognize and transport DA-BLPs to the outer membrane more efficiently than its LolCE counterpart (LoVullo et al., 2015). This prevents the toxic accumulation of BLPs in the inner membrane and subsequent cell death.

Having reviewed the three enzymes in the canonical BLP posttranslational processing pathway, we now introduce a series of enzymes recently discovered that are also involved in BLP modifications, the first of which is the lipoprotein *N*-acyl transferase system.

### Lipoprotein *N*-acyl Transferase System A and B

Triacylation is generally required for the transfer of BLPs from the inner to the outer membrane of Gram-negative bacteria via the Lol-trafficking system. As noted, this is often cited as the reason why Lnt is not essential in Gram-positive bacteria. Nonetheless, for almost a decade now, it has been known that Gram-positive *Firmicutes* possess triacylated lipoproteins, even though they lack a sequence homolog of Lnt (Asanuma et al., 2011; Nakayama et al., 2012). The mystery as to where the *N*-acylation capacity derives has recently been solved. Using a TLR-based reporter assay to screen a random transposon library, two proteins were identified in *S. aureus*, LnsA (SAOUHSC_00822) and LnsB (SAOUHSC_02761), that together perform the acylation reaction (Gardiner et al., 2020). When heterologously expressed in *Listeria monocytogenes*, which naturally only produces DA-BLPs, both LnsA and LnsB were shown to be required for the conversion of DA-BLP substrates to TA-BLP products. To date, they have been reported only in *Staphylococcal* species that make TA-Lpp. Together they form the *l*ipoprotein *N*-acyl transferase *s*ystem (LnsA and LnsB, LnsAB).

Neither LnsA nor LnsB have obvious sequence similarity with Lnt or the intramolecular transacylase, Lit (*vide infra*), the two other enzymes known to *N*-acylate BLPs at cysteine. At first pass therefore, the assumption is that LnsAB carries out the acyl transfer reaction by a mechanism that is distinct from that used by either Lnt or Lit. To date, neither the source of the acyl chain transferred by LnsAB nor the mechanism by which *N*-acylation occurs are known.

Bioinformatics analyses suggest that LnsA is the protomer in LnsAB that performs the *N*-acylation reaction (Gardiner et al., 2020). This is based on the observation that LnsA has a low sequence identity to enzymes with *N*-acyltransferase activity. Of relevance is one responsible for the formation of *N*-acyl PE from PE using phosphatidylcholine as the acyl donor. The LnsB protomer is predicted to be a membrane protein. It has weak sequence similarity with the human γ-secretase APH-1 subunit which is proposed to be responsible for chaperoning its protein substrate to this integral membrane protease. In LnsAB, it has been suggested that LnsB may play a similar role, in this instance, to guide the DA-BLPs and lipid substrates into the active site of LnsA, but other roles, catalytic or otherwise, cannot be definitively ruled out (Gardiner et al., 2020). By analogy with Lnt, LnsB may perform in the same way as has been suggested for Lnt’s TMH domain, as a membrane platform to support the nitrilase-like domain as it extends into the extracellular space, and to assist in guiding lipid and BLP substrates and products into and out of the active site (Wiktor et al., 2017).

**FIGURE 8:**
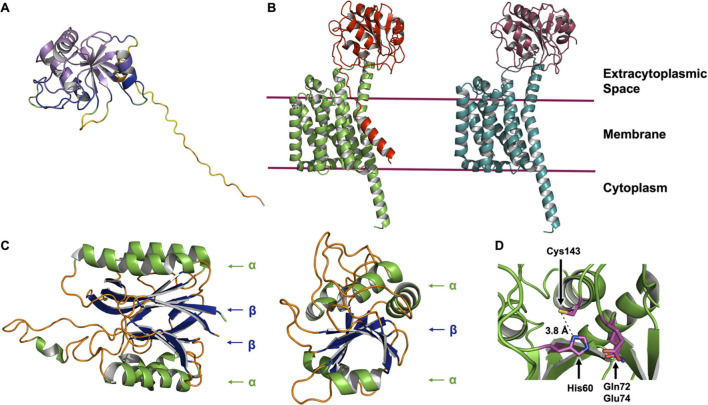
AlphaFold and ColabFold structure predictions of the LnsAB complex. **(A)** AlphaFold model of LnsA from *S. aureus*. The cartoon is colored in a rainbow based on the confidence, where red is low and violet is high confidence. The first ∼ 40 residues are modeled with low confidence and are not reliable (large unstructured yellow and orange tail). **(B)** ColabFold model of the LnsAB complex. On the left, the full LnsA sequence was used to model the complex. LnsA is colored red and LnsB green. On the right, the first 27 amino acids were deleted, mimicking the case where the predicted signal peptide in LnsA has been cleaved off by signal peptidase I. LnsA is colored in deep red and LnsB in teal. **(C)** Structure of the nitrilase-like domain in Lnt from *E. coli* (left) and the core domain of LnsA (right). Helices are colored green, β-sheets blue and unstructured regions orange. Components of the αββα sandwich fold in Lnt and the simplified αβα fold in LnsA are indicated. **(D)** View of the proposed catalytic residues. His60, Gln72, Glu74, and Cys143 are shown as sticks colored by element with magenta for carbon, blue for nitrogen, yellow for sulfur and red for oxygen. The distance between His60 and Cys143 is indicated.

The AlphaFold2 Protein Structure Database was published recently (Jumper et al., 2021). The computational method upon which it is based has received high praise and considerable media coverage for its ability to generate accurate predictions of many protein structures. The database currently contains some 365,000 structure predictions across a limited range of species which is set to grow over the coming months. While at the time of writing a structure for LnsB was not found in the database, it did contain a model for LnsA (Figure 8A). The prediction for the N-terminus (residues 1–39) of LnsA takes the form of a long, disordered region and is of low confidence. We sought a prediction for the LnsAB complex using ColabFold, and found that in this case, the N-terminus of LnsA is modeled as a transmembrane helix that packs against the transmembrane domain formed by LnsB, although at relatively low confidence (Figure 8B). An analysis of LnsA using the software SignalP 5.0 has been performed (Gardiner et al., 2020) which predicts the presence of a signal peptide and a cleavage site for a given input protein sequence. The software predicted LnsA to have a N-terminal signal peptide that is likely cleaved by signal peptidase I between residues 27 and 28 (83% probability). Accordingly, we used ColabFold to model a complex of LnsAB, where the first 27 residues of LnsA had been deleted, mimicking the final interacting species in the event the signal peptide is cleaved (Figure 8B). The LnsAB complex structure prediction in this case was almost identical to that of the complex with full-length LnsA (RMSD 0.564 Å), with the obvious exception of the missing N-terminal helix in LnsA. The interacting surfaces between the globular domain of LnsA and LnsB are conserved in both models, suggesting the signal peptide may be cleaved in the final, functional LnsAB complex. Further biochemical and structural experiments are required to validate the model, and to fully elucidate the structure and function of this novel complex.

Interestingly, the globular domain of LnsA (residues 40–189) is predicted with high accuracy to adopt a fold reminiscent of that in the nitrilase-like domain of Lnt. While the latter consists of an αββα fold, the model predicted for LnsA has just one β sheet, forming a simpler αβα domain (Figure 8C). Residues His60 and Cys143, along with one of two candidate polar residues (Gln72 or Glu73) are proposed to form the catalytic triad, based on bioinformatic analysis which characterize LnsA as a possible member of the NlpC/P60 superfamily (Gardiner et al., 2020). These residues appear to map onto the predicted structure in a way that makes reasonable biochemical sense and supports the hypothesis that LnsA houses the catalytic center in LnsAB. In the model, His60 is a nominal distance of 3.8 Å from the thiol of Cys143, and Gln72 and Glu74 are both within a reasonable distance to complete a triad, similar to that observed in Lnt (Figures 7, 8D). However, the presence of a second cysteine (Cys61), modeled only 5.6 Å from the proposed catalytic Cys143 (measured Cα to Cα), suggests the two will form a cystine within the oxidizing environment of the periplasmic space, rendering the catalytic cysteine useless. Experimental structures are clearly needed.

The identification of LnsAB represents a landmark in our understanding of the lipid-modification of BLPs in Gram-positive *Firmicutes*. Although sequence analyses and *in silico* modeling provide some indications of LnsA’s role in the *N*-acylation of BLPs, high-resolution experimental structures of LnsA and LnsB, alone and in complex with one another, coupled with mechanistic enzymology, mutagenesis, biochemical and biophysical characterization, and molecular dynamic simulations are now needed to fully appreciate how the complex forms, its stoichiometry and how it operates at a molecular level and the bases of its substrate selectivity.

### Lipoprotein Intramolecular Transacylase

The *l*ipoprotein *i*ntramolecular *t*ransacylase (Lit) enzyme generates lyso-form lipoproteins (lyso-BLPs) using DA-BLPs as substrates. Lyso-BLPs have an *N*-acyl-*S*-monoacylglyceryl-cysteine structure and were first discovered in Kurokawa et al. (2012). The *lit* gene was subsequently identified using an intergenic complementation rescue assay (Armbruster and Meredith, 2017). Lit is conserved among low-GC Gram-positive *Firmicutes*, with no homologs found in high-GC Gram-positive or in Gram-negative bacteria (Armbruster and Meredith, 2017; Armbruster et al., 2019).

**FIGURE 9:**
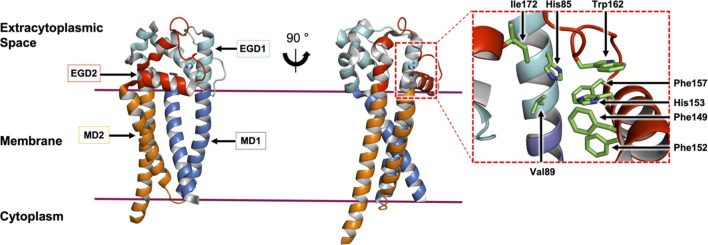
Structural features of Lit from *B. cereus.* The structure of Lit (PDP code 7B0P, Olatunji et al., 2021) is shown in two orientations viewed from the membrane plane. The protein is colored to highlight the pseudo-structural symmetry between the N-terminal membrane domain 1 (MD1) and extramembrane globular domain 1 (EGD1) (shades of blue) and the C-terminal MD2 and EGD2 (shades of red). A magnified view of the active site with conserved residues (stick representation) is shown on the right.

The crystal structure of Lit from *Bacillus cereus* was published recently (Figure 9; Olatunji et al., 2021). It is a 218 amino acid residue, cone-shaped protein with four TMHs that span the membrane. The narrow base of the cone is located within the cytoplasm and includes both the enzyme’s N- and C-termini. As expected, it is cationic. The wide end of the cone extends into the extracytoplasmic space and is covered by a dome-shaped cap domain. The latter is formed by globular sub-domains with striking shape and chemical complementarity. The cap has a hydrophobic interior that sequesters a central cavity within the TMH domain. Conserved residues map to the interface between the cap and the membrane domain, which is where the enzyme’s active site is proposed to reside. In the crystal structure, two monoolein molecules, originating from the lipid cubic phase used for crystallization, were found within the putative substrate binding pocket. These have been taken as proxies for the two acyl chains in the DA-BLP substrate of Lit (Olatunji et al., 2021).

**FIGURE 10:**
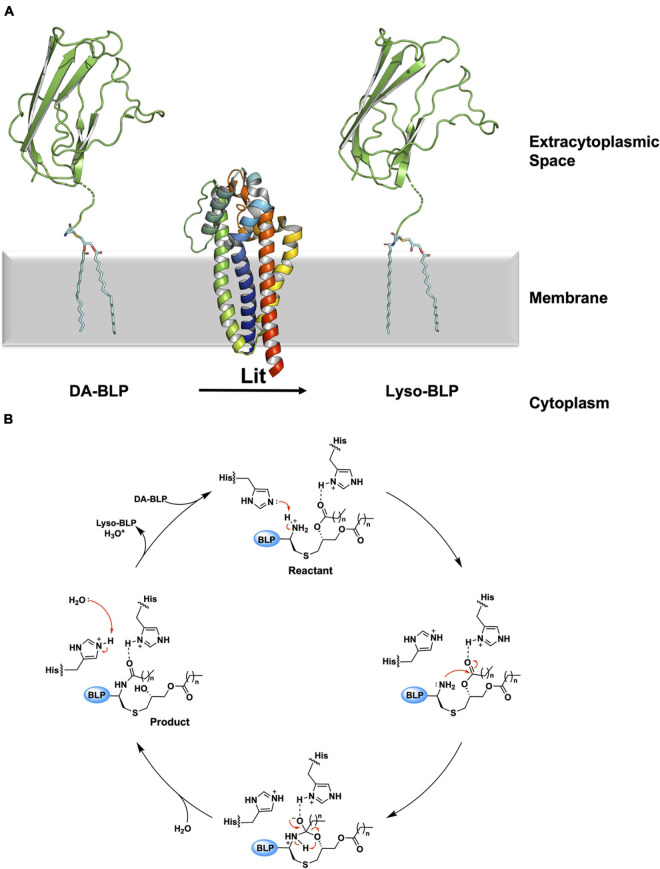
Reaction scheme for the BLP posttranslational modification carried out by Lit. **(A)** The reaction catalyzed by Lit is illustrated using solved structures of Lit from *B. cereus* (PDB code 7B0P) and of the U-domain of ICP (PDB code 2WGN), a BLP from *P. aeruginosa*. Lit catalyzes direct transfer of the acyl chain at the *sn*-2 position of the DA-BLP substrate’s glyceryl moiety to the α-ammonium group of the substrate’s N-terminal cysteine forming the lyso-BLP product. **(B)** A putative mechanism for the reaction catalyzed by a histidine dyad in Lit, adapted from Olatunji et al. (2021). Red curved arrows indicate electron flow. Double dots indicate lone electron pairs. Top: A DA-BLP substrate enters the active site. One of the catalytic histidines (His85) co-ordinates with the carbonyl oxygen of the *sn*-2 acyl chain on the substrate. The other histidine (His153) abstracts a proton from the N-terminal α-ammonium group, generating a nucleophilic amine. Right: The latter attacks the carbonyl carbon of the acyl chain at the *sn*-2 position generating an 8-membered cyclic intermediate. Bottom: Collapse of the cyclic intermediate occurs as indicated by the red arrows. Left: The lyso-BLP forms as the enzyme is restored to its original condition when a proton on the His153 imidazolium is transferred to water.

The mechanism of the intramolecular acyl transfer reaction catalyzed by Lit was investigated by mass spectrometry and nuclear magnetic resonance spectroscopy using deuterium-labeled lipopeptide and BLP substrates (Armbruster et al., 2020; Olatunji et al., 2021). These show convincingly that Lit transfers the ester-linked acyl chain at the *sn*-2 position of the glyceryl moiety on the substrate directly to the free α-amino group of its N-terminal cysteine (Figure 10A). The proposed mechanism, which does not involve the formation of an acyl-enzyme intermediate, is supported by molecular dynamics and quantum mechanics/molecular mechanics simulations (Olatunji et al., 2021). Transfer is of the general acid-base type mechanism involving two highly conserved histidine residues (His85 and His153 in *B. cereus*, Figure 10B). His153 abstracts a proton from the free ammonium group on the substrate’s N-terminal cysteine. This facilitates intramolecular attack on the ester linkage at the *sn*-2 position of the diacylglyceryl moiety leading to the formation of an 8-membered cyclic intermediate. Collapse of the latter generates the lyso-form product with one acyl chain at the *sn*-1 position of the glyceryl moiety and another in amide linkage to cysteine. The second conserved histidine (His85) remains protonated to coordinate with substrate, intermediates and the product that come and go in the course of the reaction.

DA-BLPs and TA-BLPs engage with and activate the innate immune system by binding to and signaling through TLRs. DA-BLPs are agonists of the TLR2/TLR6 heterodimer system while TA-BLPs interact with the TLR2/TLR1 pair. Crystallographic structures of TLRs in complex with diacylated and triacylated lipopeptides rationalize the nature of the BLP/TLR pairing (Jin et al., 2007; Kang et al., 2009). Specifically, the diacylglyceryl moiety of both di- and tri-acylated lipopeptides is accommodated in the relatively large hydrophobic chain binding pocket of TLR2. However, while the binding pocket in TLR1 is big enough to accommodate the chain in amide linkage at the N-terminal cysteine of the triacylated lipopeptide, the same pocket in TLR6 is blocked by two phenylalanines and is too small. Accordingly, triacylated lipopeptides preferentially bind to the TLR2/TLR1 system while diacylated lipopeptides interact with TLR2/TLR6. Lyso-form lipopeptides are also immunostimulatory as judged by measurements made with TLR2/TLR1- and TLR2/TLR6-specific reporter cell lines (Armbruster et al., 2019). Given that lyso-form BLPs have an acyl chain in amide linkage to the N-terminal cysteine it was thought they might interact preferentially with TLR2/TLR1. However, the data show preferred activation through the TLR2/TLR6 system with a signaling strength reduced by at least two orders of magnitude compared to that generated by diacylated lipopeptides. Some weak interaction with TLR2/TLR1 was recorded at high lyso-form lipopeptide levels. In consequence, Lit may have evolved in *Firmicutes*, in part, to facilitate host infection by stealth through the production of lyso-form BLPs with weak adjuvanting properties.

A second *lit* ortholog, *lit2*, was identified as part of a copper resistance mobile genetic element, alongside a second *lgt* gene and a series of copper resistance genes (a copper efflux pump, metallochaperone and copper oxidase) (Armbruster et al., 2019). This mobile genetic element is present in certain isolates of species that already possess a chromosomally encoded *lit* gene, such as *Enterococcus* sp., as well as in isolates of other Gram-positive species which lack a chromosomally encoded *lit* gene, including *Listeria monocytogenes*. The latter naturally produce DA-BLPs. When grown in the presence of 1 mM copper however, these are remodeled to lyso-BLPs. While copper is an essential nutrient, higher concentrations are toxic to bacteria, resulting in its use, and perhaps overuse, as an antimicrobial agent in the agricultural and healthcare industries. This may have provided the selective pressure that led to the evolution of the copper resistance mobile genetic element just described. While it is easy to conceive how the presence of genes encoding copper efflux pumps and metallochaperones might be advantageous in a copper-rich environment, the benefit of an *lit* gene is less obvious. In this regard, Armbruster et al. (2019) have proposed that DA-BLPs can co-ordinate copper ions through interactions with their free N-terminal α-amino group and the thioether sulfur atom of the lipobox cysteine. Upon formation of the lyso-BLP, the amino group is no longer free, resulting in a lower affinity for copper compared to its DA-BLP counterparts. This reduces the local concentration of copper at the surface of the cell membrane which results in a decreased uptake of copper into the cell. Parenthetically, some experimental evidence that DA-BLPs bind copper has been provided (Olatunji et al., 2021). In summary, a role for *lit* in AMR by ‘evading’ the host innate immune system and by providing protection against copper toxicity make it an intriguing target for the development of novel antibiotics.

### AatD

Thus far, the BLPs described in this review have been variously lipid modified at an N-terminal cysteine. Recently, a different kind of BLP has been discovered, one that bears an acyl chain in amide linkage to an N-terminal glycine. The enzyme responsible for the *N*-acylation reaction is AatD (Belmont-Monroy et al., 2020; Icke et al., 2021). It was identified based on work carried out with enterotoxigenic and enteroaggregative *E. coli* strains that cause diarrhoeal mortality and morbidity. Substrates for AatD include CexE and Aap, which are important virulence factors (Crossman et al., 2010). Both lack the conserved lipobox motif and hallmark of canonical lipoprotein processing. By contrast, these AatD substrates contain a signal peptidase I cleavage site. CexE and Aap localize to the outer leaflet of the outer membrane. Trafficking is proposed to begin with the nascent protein being inserted into the inner membrane via the SEC system. Here, the protein is anchored in place by its signal peptide that is subsequently removed by signal peptidase I. The soluble protein product with an N-terminal glycine is *N*-acylated by the action of AatD using PE as the lipid substrate. The newly formed lipoprotein, anchored in the outer leaflet of the cytoplasmic membrane by a single fatty acyl chain, is then moved across the periplasmic space and released into the outer leaflet of the outer membrane by an ATP-dependent Aat secretion system. The latter consists of five proteins (AatPABCD), the first four of which form a complex that connects the inner and outer membranes, parts of which resemble the Type I secretion system.

**FIGURE 11:**
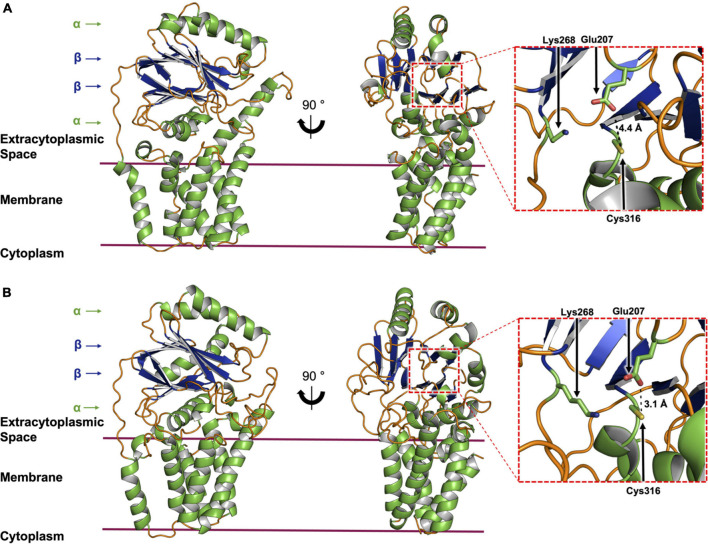
Structure of AatD from *E. coli* predicted by RoseTTAfold and ColabFold. **(A)** RoseTTAfold structure prediction for AatD. Side (left panel) and front views (middle panel) of AatD are shown with helices colored green, disordered regions orange and β-sheets blue. Arrows to the left indicate the standard αββα nitrilase-like fold that is common to Lnt and AatD. The active site is indicated by a dashed red box on the front view, with a magnified representation showing the catalytic triad (Glu207, Lys268, and Cys316) in the right panel. The distance between Glu207 and Cys316 is indicated. **(B)** ColabFold structure prediction for AatD with the same features as described in **(A)** for the RoseTTAfold model.

AatD and Lnt both perform an *N*-acylation reaction, and both work with PE as lipid substrate. However, while Lnt *N*-acylates the free amino group of a lipid-modified N-terminal cysteine, AatD catalyzes the formation of an amide linkage with the free α-amino group of an N-terminal glycine. Comparisons between AatD and Lnt show sequence homology and identity values of 20 and 10%, respectively. Despite the low sequence identity, structure prediction using the recently released RoseTTAfold algorithm (Baek et al., 2021), as well as ColabFold, provide a model for AatD that is remarkably similar to that of Lnt, with the putative catalytic triad residues organized in a near identical orientation to that seen in Lnt (Figure 11). Together these observations suggest that AatD carries out an *N*-acylation reaction in a manner similar to the reaction catalyzed by Lnt with the notable differences that (i) Lnt works with a membrane-anchored protein substrate while AatD uses one that is water-soluble, and (ii) Lnt requires a diacylglyceryl-modified cysteine at its lipoprotein substrate’s N-terminus while AatD only requires a glycine at the N-terminus of its soluble protein substrate. Experimental structures supported by biochemical and biophysical characterization are needed to establish the mode of action of this novel posttranslational modifying enzyme. Given that AatD homologs are present in pathogens such as *Enterobacterales*, *Shigella boydii, Yersinia enterocolitica*, and *Citrobacter rodentium*, and that AatD is required for the colonization of the gastrointestinal tract of murine models, this transacylase may, in time, prove to be a target for the development of antibiotics to treat infections by these pathogenic bacteria.

## Section 2: Lipoproteins and Lipoprotein Processing Enzymes: Targets for Antibiotic and Vaccine Development

### Antibiotics

As discussed, the essential nature of the lipoprotein processing pathway in Gram-negative bacteria, as well as the reliance of pathogenic Gram-positive species on the pathway for generating virulence factors, make it an important target for antibiotic development. The pathway is attractive also because mammalian systems, for the most part, lack homologous enzymes and so, off-target effects are much less likely to arise and to jeopardize drug development. Because all the enzymes involved in BLP synthesis reside in the cytoplasmic membrane with active sites facing the extracytoplasmic space, they are more accessible to administered therapeutics. For the same reason, the development of resistance through the action of drug efflux pumps is less of a concern. However, in the case of diderms, any antibiotic targeting the posttranslational processing enzymes must cross the outer membrane. It is as a result of not being able to breach this barrier, which is formidable in the case of bacteria with relatively impermeable outer membranes like *P. aeruginosa*, that many potential drugs fail to make it to the clinic (Zgurskaya et al., 2015).

Two of the enzymes reviewed in this article, Lgt and LspA, are being investigated as targets for the development of antibiotics.

### LspA Inhibitors

Globomycin and myxovirescin are natural product inhibitors that target the BLP posttranslational processing pathway at LspA. As discussed, both inhibitors have one side of their cyclic structure that is polar with a critical hydroxyl group, the so-called blocking hydroxyl, that lodges between the catalytic aspartyl residues thereby inhibiting the enzyme (Vogeley et al., 2016; Olatunji et al., 2020). Their mechanism of action is reminiscent of that described for many HIV aspartyl protease inhibitors where a non-cleavable tetrahedral intermediate analog is invoked (Brik and Wong, 2003).

Structure-activity relationships (SAR) studies of globomycin and its naturally occurring congeners identified chemical motifs that play an important role in LspA inhibition (Kiho et al., 2003, 2004). These led to the development of analogs with increased potency against Gram-negative as well as Gram-positive bacteria. Thus, analogs with longer chains at the methyl hydroxy acyl moiety had significantly improved antimicrobial activity. These observations make good physicochemical sense given how globomycin associates with LspA, with its hydrophobic side facing away from the enzyme’s binding pocket into the lipid bilayer. A longer chain would increase globomycin’s partitioning into the membrane and, in turn, increase the local concentration of the therapeutic next to the enzyme, favoring binding (Payandeh and Volgraf, 2021).

**FIGURE 12:**
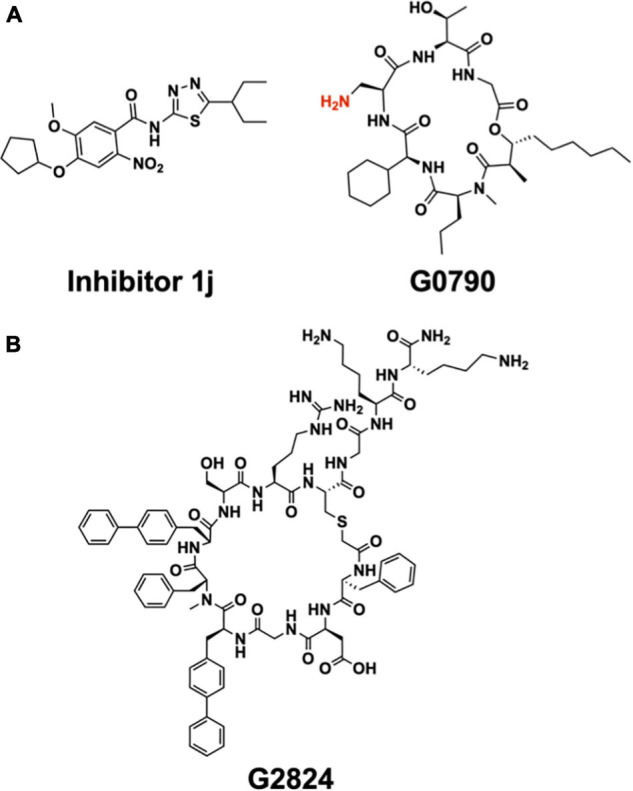
Synthetic inhibitors of LspA and Lgt. **(A)** Structure of LspA inhibitors 1j (Kitamura et al., 2018) (left) and G0790 (Pantua et al., 2020) (right). The putative blocking amine of the globomycin analog G0790 is shown in red. **(B)** Structure the macrocyclic peptide G2824 (Diao et al., 2021), an inhibitor of Lgt.

The crystal structures of LspA in complex with globomycin (Vogeley et al., 2016) has informed structure-based design of analogs, which, compared to globomycin, showed improved inhibitory activity against a uropathogenic (CFT073) strain of *E. coli* (Garland et al., 2020; Pantua et al., 2020). One of these analogs, G0796, contains (*S*)-2,3-diaminopropionic acid (DAP) in place of the serine residue in globomycin (Figure 12A). A molecular model generated for G0796 bound to LspA suggests that the analog likely inhibits the enzyme by way of its free amine, which can locate between the catalytic aspartates in the same way that the blocking hydroxyl in globomycin inhibits the protease (Pantua et al., 2020). Impressively, minimal inhibitory concentrations (MIC) were 4–8 times lower for G0796 compared to globomycin when tested against *E. coli* CFT073, *Enterobacter cloacae* (ATCC 13047), and *Klebsiella pneumoniae* (ATCC 700603).

Inhibition of *E. coli* by G0796 was found to lead to the emergence of resistance. Resistance was mediated by genomic amplification of the *lspA* gene leading to an overexpression of the enzyme and/or by deletion or mutation of the major outer membrane lipoprotein, Lpp (Pantua et al., 2020). A similar Lpp-mediated resistance and rescue of *E. coli* cell growth has been demonstrated with inhibitors targeting LspA including globomycin and myxovirescin, as well as inhibitors that block BLP transport to the outer membrane via the Lol pathway (Zwiebel et al., 1981; Yakushi et al., 1997; Xiao et al., 2012; McLeod et al., 2015; Nickerson et al., 2018). Failure to transport Lpp to the outer membrane for cross-linking to the peptidoglycan layer and stabilization of the cell envelope results from reduced or halted LspA and/or Lol system activities. This leads to immature DA-Lpp accumulating in the inner membrane where it aberrantly cross-links to the peptidoglycan layer, ultimately resulting in a malformed and malfunctioning outer membrane, and eventually to cell death. Reducing cellular levels of Lpp therefore lessens the opportunity for toxic defects to form in the cell wall and is used as a resistance mechanism to evade certain antibiotics.

A high-throughput, FRET-based peptidase assay has been used to screen a small-molecule library for compounds that inhibit LspA from *E. coli* (Kitamura et al., 2018). The substrate used was a synthetic lipobox-containing peptide, lipid-modified at the internal cysteine, and with EDANS (fluorophore) and DABSYL (quencher) chromophores at either end. The library consisted of 646,275 compounds. Screening, followed by medicinal chemistry, identified a small molecule, 1j (Figure 12A), that inhibited LspA in *in vitro* assays with an IC50 of 99 nM at an enzyme concentration of 100 nM (Kitamura et al., 2018; Kitamura and Wolan, 2018). Using 1j in combination with the outer membrane permeabilizer, polymyxin B nonapeptide, an MIC of 25 μM was recorded when tested *in vivo* with *E. coli* ATCC25922 and the membrane permeable *E. coli* DW37 strains. However, 1j was less effective in limiting the growth of *E. coli* K12 MG1655, the clinical *E. coli* isolate UTI89 as well as other bacteria tested including *P. aeruginosa Klebsiella pneumoniae* (*Yersinia pestis*, *Staphylococcus epidermidis* and *S. aureus*, Kitamura et al., 2018).

A recombinant ppBLP converted by Lgt to pBLP has been used as a substrate with which to monitor LspA activity in a gel-shift assay (Vogeley et al., 2016; Olatunji et al., 2020). Interestingly, the results obtained for globomycin inhibition with the gel-shift and with FRET-based assays of the type just described were found to depend on the LspA ortholog used. Specifically, similar IC50 values, close to half the enzyme concentration used for assay, were observed with LspA from *P. aeruginosa* and *S. aureus* in the FRET assay. By contrast, in the gel-shift assay globomycin had very little inhibitory effect on LspA from *S. aureus* (estimated IC50, 170 μM, at an enzyme concentration of 0.5 μM) while the *P. aeruginosa* ortholog was fully inhibited at single digit micromolar globomycin concentrations (IC50, 0.6 μM at an enzyme concentration of 0.5 μM). These profound differences in behavior depending on whether a more natural as opposed to a simpler, synthetic substrate is used, highlight the importance of not relying on a single assay type to characterize enzymes and to screen for inhibitors.

### Lgt Inhibitors

Attention has recently turned to Lgt, the first enzyme in the canonical BLP posttranslational processing pathway, as a target for the development of antibiotics (Diao et al., 2021). Considerable progress has already been made based on a screen of a macrocyclic peptide library generated by the mRNA-based RaPID (random non-standard peptides integrated discovery) method. Two peptides, G2824 and G9066, proved particularly interesting, inhibiting Lgt from *E. coli* in *in vitro* assays with IC50 values of 180 and 240 nM, respectively (Figure 12B). Unfortunately, their performance in *in vivo* assays of the growth of a clinical uropathogenic *E. coli* strain was not as impressive, with MIC values of approximately 100 μM. However, when used in combination with treatments that permeabilize the outer membrane, such as supplementation with EDTA or the *imp4213* allele of *lptD*, the MIC values dropped into the single digit micromolar range.

Interestingly, deletion of Lpp contributed to a reduction in MIC values (to 30–60 μM). This is in distinct contrast to the behavior observed with LspA, where reducing Lpp levels provided resistance to LspA inhibitors. An explanation for the disparate behaviours of Lgt and LspA inhibition takes the following form. When LspA is inhibited, Lpp accumulates in the inner membrane where it cross-links to the peptidoglycan layer with toxic consequences. Lowering Lpp levels, by whatever means, reduces the toxic Lpp-peptidoglycan load, and rescues cell growth. By contrast, when Lgt is inhibited, while the immature prepro-form of Lpp accumulates in the inner membrane, it does not cross-link to the peptidoglycan layer, unlike its lipidated pro-Lpp counterpart, and therefore it does not cause the cell wall to malform in a toxic manner. In this way then, targeting Lgt makes good sense since the bacteria cannot exploit deletion or mutation of the *lpp* gene as a resistance mechanism.

To understand the mechanism of action of these cyclic peptide inhibitors, Diao et al. (2021) examined the effect that a titratable, inducible Lgt protein depletion had on the morphology, growth and survival of *E. coli*. Reducing Lgt to 75% of wild-type levels was enough to kill the cells. A lesser reduction to just 90% of normal levels resulted in an increase in complement-mediated killing of a strain that is normally serum-resistant. A significant change in cell morphology upon Lgt deletion was also observed. This was evidenced by an increase in cell size, a more rounded cell shape, and enhanced outer membrane blebbing. These changes were accompanied by an increased sensitivity to antibiotics. Specifically, the MIC for vancomycin dropped from over 100 μM in wild-type *E. coli* to 12.5 μM in cells with 90% of the usual Lgt content. Similar trends were reported with rifamycin, penicillin G, oxacillin, zeocin, and norfloxacin. These findings suggest that Lgt inhibitors are ideally suited for use in a combination antibiotic therapy approach. In this type of application, they would function to permeabilize the outer membrane for easier access to partner antibiotic targets residing in the periplasm, the inner membrane or the cytoplasm.

### Vaccines

To date, antibiotic development in the BLP arena has focused on the enzymes involved in posttranslational processing. Because BLPs take up residence in extracellular locations, they are ideal candidates for interrogation, identification and attack by the adaptive immune system. This same logic forms the basis for vaccine development where antibodies are raised to react against pathogenic bacteria that harbor antigenic proteins on their cell surface. Thus, lipoproteins such as Aap and CexE, in the outer leaflet of the outer membrane of Gram-negative cells are suitably located to trigger an immune response leading to antibody production. Similarly, BLPs in monoderms, which reside in the outer leaflet of the cytoplasmic membrane, are favorably positioned to elicit an immune response.

In the case of BLPs, the U-domain of the lipoprotein is the antigen. Interestingly, the lipid modification in combination with the N-terminus of mature BLPs engage with the innate immune TLR system where they behave as fast-acting and potent adjuvants. The motif is referred to as a pathogen- or a microbial-associated molecular pattern (P/MAMP). As adjuvants, MAMPs eventually activate the slower responding adaptive arm of the immune system to generate antibodies specific to the antigen. Adjuvants, such as aluminium oxide (alum), are often included as stand-alone components to elicit a faster, stronger and longer lasting immune response to antigens in vaccine formulations. However, when included as individual additives, their effectiveness is lessened. This is, in part, because the different components can diffuse away from one another at the site of vaccination, or in some cases, adjuvants can damage the antigen.

**FIGURE 13:**
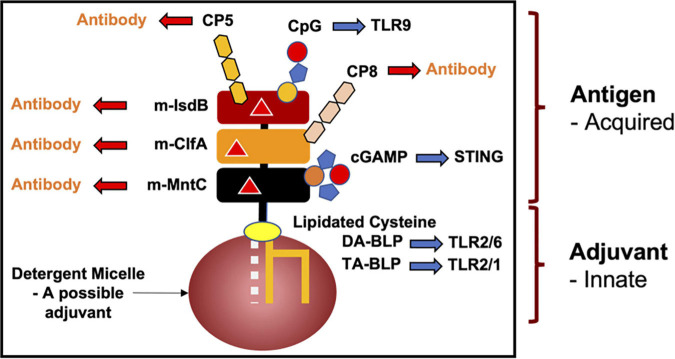
A hypothetical recombinant or synthetic monomolecular self-adjuvanting multivalent super-antigen modeled on a BLP for use in the development of vaccines for the prevention of a variety of ailments. The bottom portion of the model vaccine includes the BLP MAMP, with two or three fatty acyl chains. Respectively, they represent potent adjuvanting agonists of the innate immune system which signal through the TLR2/TLR6 or TLR2/TLR1 heterodimers. The upper portion of the vaccine includes, in this instance, three different contiguous protein or peptide antigens that engage with the acquired immune system for antibody production. Synthetic peptide antigens lend themselves to chemical modification with ligands such as cyclic guanosine monophosphate-adenosine monophosphate (cGAMP), cytosine monophosphate guanosine (CpG), and serotype 5 or serotype 8 *Staphylococcal* capsular polysaccharide (CP5 and CP8), that couple with the innate and acquired immune systems as indicated. Here, STING refers to the *St*imulator of *in*terferon *g*enes, a signaling cascade that triggers an immune response. Glycosylation at permissive sites in target proteins might be introduced by recombinant glycobiology. ‘Super’ vaccines can be designed to generate antibodies that will cater to seasonal strains, for example, by varying the identity of the amino acid/s at strategic sites in the antigen [red triangle in the individual peptide or protein antigens representing a mutation (m)]. In this example, the vaccine is solubilized in a detergent micelle. Alternative dispersion and delivery systems might include bilayered lipid vesicles. The detergents and lipids in the delivery systems can also have valuable adjuvanting properties. m-MntC, m-ClfA, and mlsdB refer, respectively, to a domain or a sequence containing a linear or a conformational epitope from the manganese ABC transporter subunit C, the clumping factor and the iron regulated surface determinant scavenging protein in *S. aureus*. The prefix ‘m’ denotes the presence of sites where residue variability can be introduced for ‘super’ vaccine generation.

Because lipoproteins are part MAMP, it should be obvious that a mature BLP, with a U-domain that is antigenic, will have built-in, self- or auto-adjuvanting properties. Thus, antibodies will be produced against the BLP’s antigenic U-domain in a process that is enhanced (adjuvanted) by the MAMP covalently attached at its N-terminus. BLPs are therefore attractive candidates for vaccine development, not only for their natural self-adjuvanting properties, but also because they can be produced and modified, essentially to order, by means of recombinant DNA technology and synthetic organic chemistry. While the focus of this review is on pathogenic bacteria, the concept of a self-adjuvanting vaccine can be generalized to encompass the prevention of pathogenic viral and eukaryotic infections, food and environmental allergies, and cancers (Figure 13).

In what follows, we provide three examples of BLPs that have been considered or are being used as self-adjuvanting vaccines.

The first is the outer surface protein A (OspA) from *Borrelia burgdorferi*. The full-length, lipidated OspA was used in the original recombinantly expressed BLP approved as a vaccine against Lyme disease, where ticks are the vector (Steere et al., 1998). The vaccine, licensed as LYMErix by SmithKline Beecham (now GlaxoSmithKline), has an interesting mode of action. Upon vaccination, antibodies against OspA are produced. When a tick that is infected with *Borrelia burgdorferi* takes a blood meal from an immunized individual, the blood borne antibodies make their way to the tick’s stomach where the bacteria are eliminated before even entering the person. LYMErix was effective in reducing the incidence of Lyme disease by 76%. Unfortunately, cases of severe arthritis in a subset of vaccinated individuals led to the vaccine being withdrawn from the market. Some speculated this adverse side effect was due to the similarity of the U-domain of OspA to a human protein, lymphocyte function-associated antigen (Nigrovic and Thompson, 2007; Willyard, 2014). Regardless, this represents an example where the antigenic U-domain of a BLP works in concert with an inbuilt PAMP in the form of a lipidated N-terminus to elicit an effective immune response and protection against a pathogenic organism.

The second example of a self-adjuvanting BLP-based vaccine is one that targets *Neisseria meningitidis*, an encapsulated relative of *N. gonorrhoeae*, which can cause meningococcal disease. Protection against several strains of the bacteria is available through vaccines based on the polysaccharide component of its protective capsule. *N. meningitidis* strain B is a notable exception. The capsular polysaccharide of the B strain is identical to the post-translational polysialylated modifications made to a human protein (Human Fetal Neural Cell Adhesion Molecule), and so does not elicit an immune response. As an alternative, vaccines have been developed against *N. meningitidis* B based on one of its BLPs. Specifically, factor H binding protein (fHbp), a surface exposed TA-BLP. Immunization with triacylated-fHbp induced serum bactericidal antibody production and conferred protective immunity against *N. meningitidis* B (Brendish and Read, 2015). In 2015, Pfizer concluded clinical trials and received FDA approval for their *N. meningitidis* B vaccine, Trumenba, the second recombinant BLP licensed for use in human vaccines. Trumenba contains two variants of fHbp, both of which are lipidated at the N-terminus, undoubtedly there to take advantage of the antigen’s self-adjuvanting features (Gandhi et al., 2016; Luo et al., 2016).

The final example of a vaccine that utilizes BLPs is Pfizer’s 4-antigen *S. aureus* vaccine, SA4Ag (Begier et al., 2017). SA4Ag includes the U-domain (unlipidated) of a recombinant BLP, the manganese transporter C (MntC), two capsular polysaccharide conjugates (serotypes 5 and 8, CP5 and CP8), and the recombinant surface protein clumping factor A (ClfA). To date, no vaccine is currently available to prevent or treat *S. aureus* infections. Previous attempts to develop vaccines showed promising preclinical serology with murine sepsis models which failed to translate into clinical efficacy. Unfortunately, Pfizer’s tetravalent SA4Ag vaccine proved no different. Despite strong performance in pre-clinical mouse studies, as well as phase 1 and early phase 2 clinical trials showing robust immune responses to the vaccine, late phase 2 trials showed little difference between individuals given placebo versus vaccine. With no clinical efficacy, development of the SA4Ag has been abandoned by the company (Scully et al., 2021). Regardless, the potential of MntC, which is one of the most abundant BLPs in *S. aureus* and is required for virulence in methicillin-resistant strains (Diep et al., 2014; Nguyen and Götz, 2016), as an effective vaccine component should not be lost sight of. Its U-domain robustly produced protective immunity against infection by *S. aureus* and *S. epidermidis* in mice. Potentially, after a concerted effort to optimize modifications, such as the inclusion of a lipidated N-terminus to stimulate innate responses in humans, the protective immunity seen in mice may translate into clinical efficacy.

## Concluding Remarks

Since the identification of Braun’s lipoprotein (Lpp) in *E. coli* in 1969 (Braun and Rehn, 1969; Asmar and Collet, 2018), a concerted effort has led to a deeper understanding of the physiological role of this class of bacterial proteins and to a structural and functional understanding of the biosynthetic pathways that produce them. The ability to isolate BLPs in their native form from inner and outer membranes, and to characterize the structure of their lipid components using powerful analytical methods such as NMR and mass spectrometry, have contributed to a fuller appreciation of the processes by which they are posttranslationally modified and trafficked in the cell.

Advances in recombinant membrane protein expression and purification, combined with the application of new structural biology methods, have proven crucial in deciphering the molecular mechanisms of the enzymes involved in BLP maturation. Of note, the lipid cubic phase crystallization method was used to obtain high-resolution structures of LspA, Lnt and Lit. Structures of Lgt and Lnt have been determined by more traditional vapor diffusion crystallization. While the structures of LnsAB and AatD remain unsolved experimentally, recent advances in the algorithms used for structure prediction can now provide computational models with valuable insights into mechanism of action and drug development. Of course, this must be followed up on by the provision of high-resolution experimental structures.

Sadly, structures of full-length BLPs are few and far between. One of the earliest appeared as the cytochrome subunit in the photosynthetic reaction center from *Blastochloris viridis* in 2012 (Roszak et al., 2012). Relatedly, the first structure of this reaction center resulted in the Nobel Prize in Chemistry awarded to Michel, Deisenhofer and Huber in 1988. Other full-length structures have appeared since, notably in multi-subunit complexes solved by single particle cryo-electron microscopy (Sun et al., 2018; Sharma et al., 2021; Tang et al., 2021).

Biochemical characterization of the BLP posttranslational modifying enzymes benefited enormously from the development of functional assays using purified recombinant lipoproteins and natural and synthetic lipopeptides. These same functional assays were exploited in structure-activity relationship studies of natural and synthetic inhibitors of these enzymes and in trials to screen for new molecules with improved drug-like properties.

Recent evidence suggests that in addition to playing a critical role in the proper physiological functioning of cells, BLPs contribute to facilitating how bacteria adapt to their environment. Examples include the involvement of BLPs in adapting to toxic concentrations of metal ions, such as copper, and in drug efflux. Further, at least in the case of commensals and pathogenic bacteria, BLPs play an active role in host immune homeostasis. How the lipid profile of BLPs selectively triggers innate and subsequent adaptive immune responses, and how this might be exploited prophylactically and therapeutically are areas that are ripe for development.

Despite the advances in our understanding of BLPs and how they are produced, much of the work done to date has been carried out with BLPs, lipopeptides and enzymes in isolation under *in vitro* conditions. Accordingly, we know very little about interactions with and between BLPs that presumably take place in the cell membrane during the different phases of growth. *E. coli*, for example, has over 80 different BLPs. One of them, Lpp, is present to the extent of up to a million copies per cell. Processing of BLPs therefore must be extremely efficient. Indeed, it has been suggested that efficiency may come about through channeling interactions between processing enzymes in the membrane. At the same time, processing must be highly specific in terms of the reactions catalyzed but promiscuous regarding the many and diverse BLP substrates with which the corresponding enzymes act.

The last decade has witnessed significant advances in the BLP research arena. High-resolution structures of the three enzymes involved in the canonical PTM of Gram-negative BLPs, Lgt, LspA, and Lnt, were determined, allowing a better understanding of their catalytic mechanism. The mystery of how TA-BLPs are produced in *S. aureus* has been solved with the identification of the LnsAB system. Lit, the enzyme responsible for the production of lyso-form BLPs has been identified, its structure has been determined to high resolution, and its mechanism of action has been established. Yet to be determined is the synthetic origin and the function of two BLP types, one with an acetyl and the other with a dipeptide *N*-modification at the diacylglyceryl-cysteine, identified by Kurokawa et al. (2012). Very recently, a new type of lipoprotein, one that is *N*-acylated at its N-terminal glycine, as well as the enzyme responsible for its synthesis, AatD, have been discovered. Thus, while we know a lot about BLPs, there remains much to understand concerning their structure and function, their regulated synthesis and trafficking, and where they reside in the cell under normal and stress conditions. The role they play in host-pathogen and host-commensal communication is another area about which we know little. Filling in these gaps in knowledge is a worthy endeavor that will provide a deeper understanding of and control over this important group of biological macromolecules that are of great physiological, medical and biotechnological significance.

## Author Contributions

LS, SO, and MC researched, wrote and edited the manuscript. All authors contributed to the article and approved the submitted version.

## Conflict of Interest

The authors declare that the research was conducted in the absence of any commercial or financial relationships that could be construed as a potential conflict of interest.

## Publisher’s Note

All claims expressed in this article are solely those of the authors and do not necessarily represent those of their affiliated organizations, or those of the publisher, the editors and the reviewers. Any product that may be evaluated in this article, or claim that may be made by its manufacturer, is not guaranteed or endorsed by the publisher.

## Abbreviations

AatD: glycine *N*-acyl transferase from the AatPABCD secretion system
AMR: antimicrobial resistance
BLP: bacterial lipoprotein
DA-BLP: diacylated-bacterial lipoprotein
Lgt: lipoprotein diacylglyceryl transferase
Lit: lipoprotein intramolecular transacylase
LnsA: lipoprotein *N*-acyl transferase system A
LnsB: lipoprotein *N*-acyl transferase system B
Lnt: lipoprotein *N*-acyl transferase
Lol: localization of lipoproteins
Lpp: Braun’s lipoprotein
LspA: lipoprotein signal peptidase
MIC: minimum inhibitory concentration
MAMP: microbial associated molecular pattern
pBLP: probacterial lipoprotein
PE: phosphatidylethanolamine
PAMP: pathogen associated molecular pattern
PG: phosphatidylglycerol
ppBLP: preprobacterial lipoprotein
PTM: posttranslational modification
Sec: secretory translocase
TA-BLP: triacylated-bacterial lipoproteins
TAT: twin-arginine translocase
U-domain: ultra-domain.
